# Wide-coverage relation extraction from MEDLINE using deep syntax

**DOI:** 10.1186/s12859-015-0538-8

**Published:** 2015-04-01

**Authors:** Nhung TH Nguyen, Makoto Miwa, Yoshimasa Tsuruoka, Takashi Chikayama, Satoshi Tojo

**Affiliations:** 1 School of Information Science, Japan Advanced Institute of Science and Technology, Ishikawa, Japan; 2 Toyota Technological Institute, Nagoya, Japan; 3 Graduate School of Engineering, The University of Tokyo, Tokyo, Japan

**Keywords:** Predicate-argument structures, Biomedical relation extraction, Open information extraction

## Abstract

**Background:**

Relation extraction is a fundamental technology in biomedical text mining. Most of the previous studies on relation extraction from biomedical literature have focused on specific or predefined types of relations, which inherently limits the types of the extracted relations. With the aim of fully leveraging the knowledge described in the literature, we address much broader types of semantic relations using a single extraction framework.

**Results:**

Our system, which we name PASMED, extracts diverse types of binary relations from biomedical literature using deep syntactic patterns. Our experimental results demonstrate that it achieves a level of recall considerably higher than the state of the art, while maintaining reasonable precision. We have then applied PASMED to the whole MEDLINE corpus and extracted more than 137 million semantic relations. The extracted relations provide a quantitative understanding of what kinds of semantic relations are actually described in MEDLINE and can be ultimately extracted by (possibly type-specific) relation extraction systems.

**Conclusion:**

PASMED extracts a large number of relations that have previously been missed by existing text mining systems. The entire collection of the relations extracted from MEDLINE is publicly available in machine-readable form, so that it can serve as a potential knowledge base for high-level text-mining applications.

**Electronic supplementary material:**

The online version of this article (doi:10.1186/s12859-015-0538-8) contains supplementary material, which is available to authorized users.

## Background

The increasing amount of scientific articles in the biomedical domain leads to a growing demand from biologists to access information in the literature in more structural form [1]. This demand motivates many researchers and scientists to work on *relation extraction*, an information extraction task that attempts to extract semantic relations between important biomedical concepts. Most of the previous work on relation extraction from biomedical literature focuses on specific or predefined types of relations, such as protein-protein interactions [2-5], protein-gene interactions [6], drug-drug interactions [7], drug-disease treatment [8], and biomolecular events [9]. The types of relations that can be extracted by existing approaches are, therefore, inherently limited.

Recently, an information extraction paradigm called Open Information Extraction (OIE) has been introduced to overcome the above-mentioned limitation [10-12]. OIE systems aim to extract all triples consisting of argument phrases (arg1, arg2) from the input sentence and a relational phrase (rel) that expresses the relation between arguments, in the format of (arg1; rel; arg2). OIE systems that have been developed so far include TextRunner [10], ReVerb [11], and OLLIE [12]. They first identify relation phrases by using part-of-speech patterns and syntactic and lexical constraints, and then detect arguments by some heuristics. Recently, advanced OIE systems have been built to tackle nominal relations [13] and *n*-ary relations [14]. Although the concept of OIE is certainly appealing, our preliminary experiments using ReVerb and OLLIE have suggested that these state-of-the-art OIE systems for the general domain do not perform well on biomedical text.

This observation has motivated us to develop PAS-MED, a wide-coverage relation extraction system for biomedical text. Our system uses Predicate-Argument Structure (PAS) patterns to detect the candidates of possible biomedical relations. A PAS is composed of a predicate and its arguments and describes (shallow) semantic relationships between words in a sentence. For example, the sentence “Macrophages are activated by LPS” has a PAS consisting of the predicate ‘activate’ and its two arguments ‘LPS’ (subject) and ‘macrophages’ (object). We decided to use PAS patterns because they are well-normalized forms that represent deep syntactic relations. In other words, multiple syntactic variations are reduced to a single PAS, thereby allowing us to cover many kinds of expressions with a small number of PAS patterns.

Using PASs has been a practical approach to domain-independent information extraction. Some annotated corpora of PAS frames in general domains such as PropBank [15], VerbNet [16], and FrameNet [17] have been published for the research community. BioProp [18] and PASBio [19] are PAS frames for the biomedical domain based on PropBank. BioProp contains 2382 predicates for 30 biomedical verbs. PASBio includes the analyzed PASs of 30 verbs describing molecular events.

Syntactic structures of the types other than PASs have also been employed in biomedical relation extraction [6,8,20,21]. Rinaldi et al. [20] introduced three levels of patterns to detect protein-protein interactions in the GENIA corpus. The first level is syntactic patterns that capture some important syntactic phenomena (e.g. active, passive, nominalizations). Next, they combined different syntactic patterns to create a semantic rule. On the third level, the semantic rules were combined with lexical and ontological constraints to obtain specialized queries that can detect a domain-specific relation. RelEx [6] also used a pattern-based approach to extract protein-gene interactions. The patterns include three crafted rules constructed based on the dependency parse tree of a sentence.

Perhaps the most similar and relevant to our work is SemRep [22,23] and the system by Nebot and Berlanga [24]. SemRep is a rule-based semantic interpreter that extracts semantic relationships from free text. Their relationships are represented as *predications*, a formal representation consisting of a predicate and arguments. SemRep extracts 30 predicate types, mostly related to clinical medicine, substance interactions, genetic etiology of disease and pharmacogenomics. Their predicates were created by modifying 30 relation types of the UMLS Semantic Network [25]. The system by Nebot and Berlanga [24] extracts explicit binary relations of the form <*subject, predicate, object*> from CALBC initiative [26]. To detect candidate relations, they proposed seven simple lexico-syntactic patterns. These patterns are expressed in part-of-speech tags in which relational phrases reside between the two entities.

We have designed PASMED with a particular focus on recall, in regard to its extraction performance. This is primarily because we wanted to extract all binary relations between important biomedical concepts described in the whole MEDLINE. The use of PAS patterns helped us to achieve relatively high recall (while keeping reasonable precision), because PAS patterns effectively represent many lexico-syntactic patterns at an abstract level and thus are robust to various syntactic transformations such as passivization, control constructions, relative clauses, and their combinations, which are quite common in sentences expressing biomedical relations. To the best of our knowledge, this is the first time that a PAS-based approach has been applied to the entire MEDLINE and evaluated in terms of open-domain relation extraction.

In this article, we first describe details about our PAS patterns and the extraction model employed by PASMED. We then briefly explain our guideline of manually evaluating the extracted relations. The second half of the article is devoted to presenting and discussing results of our system, its comparison with other systems, its limitation and the output of the whole MEDLINE. Finally, we conclude our work and propose some future directions.

## Methods

Our system uses a set of PAS patterns to detect the candidates of semantic relations. First, Mogura [27], a high-speed version of the Enju parser [28], is employed to extract NP pairs that satisfy predefined PAS patterns from sentences. Next, named entities in the NP pairs are identified by MetaMap [29]. Because MetaMap uses string matching to map biomedical texts to the concepts in the UMLS Metathesaurus [30], its output contains many spurious entities. In order to remove false positives, we conduct post-processing using information on parts-of-speech and frequencies of entities. Finally, a relation between two entities is extracted if and only if the pair of semantic types is included in the UMLS Semantic Network [25].

### Crafting PAS patterns

Since we attempt to extract unrestricted types of relations, there is no labeled corpora suitable for training a machine-learning based extraction model. We therefore took a practical approach of creating PAS-based extraction patterns manually by observing actual linguistic expressions. We decided to use PASs in this work primarily because PASs are a viable formalism for building shallow semantic representations of biomedical verbs [31]. As a result of recent advances in parsing technology, there are now publicly available deep parsers that can output PASs and are both scalable and accurate. The Enju parser is one of those parsers and has shown to be one of the most accurate syntactic parsers for biomedical documents [28].

In order to find appropriate PAS patterns, we have first observed textual expressions that represent biomedical relations in the GENIA corpus [32] and found that those relations are usually expressed with verbs and prepositions. Examples of those are *E* *n* *t* *i* *t* *y*_*A*_ *{affect, cause, express, inhibit...}* *E* *n* *t* *i* *t* *y*_*B*_ and *E* *n* *t* *i* *t* *y*_*A*_ *{arise, happen,...} {in, at, on...} Location*. Based on these observations, we create patterns that consist of three elements: (1) NP _1_ containing *E* *n* *t* *i* *t* *y*_*A*_, (2) NP _2_ containing *E* *n* *t* *i* *t* *y*_*B*_ and (3) a verbal or prepositional predicate that has the two NPs as arguments. Our patterns in predicate-argument form and their corresponding examples are presented in Table 1. It should be noted that no sentences in the GENIA corpus, which we examined for developing these patterns, were used in our evaluation experiments.

**Table 1:** **Our PAS patterns focus on verb and preposition predicates**

**No.**	**PAS Patterns**	**Examples**
1	*N* *P*_1_←**Verb**→*N* *P*_2_	Protein RepA(cop) ← affects → a single amino acid
2	*N* *P*_1_←**Verb**→*b* *y*+*N* *P*_2_	Diabetes ← induced → by streptozotocin injection
3	*N* *P*_1_←**Verb**→*N* *P* ^′^	Endothelin-1 (ET-1) ← had → a strong effect
	$\overset {\uparrow }{Prep.} \rightarrow {NP}_{2}$	$\overset {\uparrow }{in}$ → all trabeculae
4	*N* *P*_1_←**Link. Verb**→*A* *D* *J* *P*←*P* *r* *e* *p*.→*N* *P*_2_	EPO receptor ← be → present ←*i* *n*→ epithelial cells
5	*N* *P*_1_←**Verb**←*P* *r* *e* *p*.→*N* *P*_2_	Apoptosis ← involved ← in → CD4 T lymphocytes
6	*N* *P*_1_←**Prep.**→*N* *P*_2_	vitronectin ← in → the connective tissue

An arrow going from *a* to *b* means that *a* modifies *b*, where *a* is called a predicate, and *b* is called an argument. <*N* *P*_1_,*N* *P*_2_> is a relevant NP pair in each pattern.

Pattern 1 and 2 capture expressions of transitive verbs in active and passive voices respectively. Their relevant NP pairs consist of subjects and objects of the verbs. Pattern 3 deals with verbal structures in which transitive verbs modify a noun phrase to present specific actions, e.g., ‘play a role’ and ‘produce changes’. Pattern 4 is used for linking verbs. A linking verb modifies an adjective. Hence, if a noun phrase related to the adjective is found, the phrase and the subject of the verb form a relevant NP pair. To deal with intransitive verbs, we use Pattern 5. An intransitive verb has no direct object, but it can be modified by a prepositional phrase to describe the action in detail. In this case, the prepositional phrase and the subject of the verb constitute a relevant NP pair. The final pattern (Pattern 6) is used for prepositions, which would capture localization and whole-part relations.

The elements *N* *P*_1_ and *N* *P*_2_ in each pattern shown in Table 1 are used to create candidates of our relation extraction step.

In order to estimate the coverage of our patterns, we applied them to three protein-protein interaction (PPI) corpora (AIMed, BioInfer and LLL [3,33]), two drug-drug interaction (DDI) corpora (MedLine and DrugBank [7]), and the GENIA corpus [32]. We then checked if the entities in the annotated relations are included in the NP pairs of our patterns. For instance, according to the AIMed corpus, there is a PPI between ‘IFN-gamma’ and ‘IFN-alpha’ in the sentence “Levels of IFN-gamma is slightly increased following IFN-alpha treatment". This PPI is covered by Pattern 2, in which *N* *P*_1_ is ‘Levels of IFN-gamma’ and *N* *P*_2_ is ‘IFN-alpha treatment’.

The results in Table 2 show that the patterns cover over 80% of the entities in the GENIA events and PPIs of the LLL corpus sufficiently. This is somewhat expected since our PAS patterns are created based on the observations on the GENIA corpus and the LLL corpus contains only 50 sentences. However, for the other cases, our patterns only cover a small portion, e.g., 46% relations of the BioInfer, and 53% of the AIMed. Relations that our patterns miss can be categorized into two groups: (1) nominal relations, e.g., ‘CD30/CD30L interaction’, and (2) relations that need other information, such as coreference resolution, to be inferred. These kinds of relations are hard to identify by only using a pattern-based approach and are left for future work.

**Table 2:** **Expected recall of our PAS patterns on various corpora**

**PPI**			**DDI**		**GENIA**
**AIMed**	**BioInfer**	**LLL**	**MedLine**	**DrugBank**	
53%	46%	82%	64%	62%	80%

### Extracting semantic relations

Named entity recognition (NER) is an important text processing step that needs to be performed before relation extraction. Most of previous machine-learning NER tools have focused on detecting gene/protein names [34], gene/protein, cell line and cell type [35], drugs and chemicals [36]. Those tools perform well with the targeted entities but it is not easy to extend them to other types of entities. Moreover, they only locate entities in text and do not offer other information such as global identifiers (IDs) of the recognized entities, which will be useful for linking them with information stored in biomedical databases. In this work, we use MetaMap [29], a dictionary-based tool that maps biomedical texts to the concepts in the UMLS Metathesaurus [30].

The Metathesaurus is a large database that contains biomedical and clinical concepts from over 100 disparate terminology sources. In order to integrate them into a single resource, a unique and permanent concept identifier (CUI) is assigned to synonymous concepts or meanings [37]. For instance, the Metathesaurus maps the two strings of ‘Chronic Obstructive Lung Disease’ from Medical Subject Headings (MSH) and ‘COLD’ from National Cancer Institute thesaurus (NCI) to a concept whose CUI is ‘C0009264’. By using MetaMap, we can obtain the CUI and the source names of an entity. Although MetaMap does not perform as well as machine-learning tools in terms of recognition accuracy, it meets our requirement of detecting every entity in texts and outputs the Metathesaurus CUI, i.e., a global ID for each entity.

Since MetaMap uses string matching techniques to identify entities, it generates many false positive entities. We apply two post-process steps to remove these entities from MetaMap’s output. In the first step, we remove all entities that are verbs, adjectives, prepositions or numbers because we are only interested in noun or noun phrase entities. The second step is used to avoid common noun entities, e.g., ‘study’, ‘result’ and ‘relative’. We first construct a dictionary of named entities based on MetaMap’s results of the whole MEDLINE [38] and remove highly frequent entities from it. This dictionary is then used to check the validity of named entities.

To evaluate the effectiveness of these post-processing steps, we conducted a small set of experiments using several annotated corpora. We employed MetaMap to detect proteins in AIMed, BioInfer and LLL [3,33], and drugs in the SemEval-2013 task 9 corpus [7]. We then post-processed these outputs and compared them with labeled entities to evaluate the performance of our post-processing. The scores in Table 3 show that our filtering improved the F-scores significantly for both proteins and drugs, resulting in F-scores of 51.37% on proteins and 71.38% on drugs. This performance is comparable to that of CubNER, an unsupervised NER tool for biomedical text [39].

**Table 3:** **Performance of our post-processing on proteins and drugs detection**

**Protein**	**Acc.**	**Pre.**	**Re.**	**F. (%)**
MetaMap	58.10	15.72	63.21	25.18
After filtering	***88.93***	***55.77***	47.61	***51.37***
**Drug**				
MetaMap	62.61	20.86	79.51	33.04
After filtering	***93.96***	***83.26***	62.47	***71.38***

These scores were generated by using the evaluation script of CoNLL 2000.

We obtain named entities in candidates of NP pairs after our post-processes. Next, each entity in *N* *P*_1_ is coupled with every entity in *N* *P*_2_ to create a candidate of semantic relation. It should be noted that separate entities inside a noun phrase are not considered to constitute a relation. We then use the UMLS Semantic Network as a constraint to filter out relations that are likely to be spurious. More specifically, the Semantic Network provides a relation ontology that consists of a set of relations between semantic types, such as relations between ‘Gene or Genome’ and ‘Enzyme’, or ‘Hormone’ and ‘Disease or Symptom’. We check if the pair of semantic types of the two entities in a candidate exists in the ontology or not. If it does, the candidate is included in the output of the system; otherwise, we reject it.

Our process can be described formally as follows. Let us denote by <*N* *P*_1_,*N* *P*_2_> a relevant NP pair, by *e*_1*i*_ (*i*=1,2,...) entities in *N* *P*_1_, and by *e*_2*j*_ (*j*=1,2,...) entities in *N* *P*_2_. Every pair of entities <*e*_1*i*_,*e*_2*j*_> can compose a candidate of semantic relation. Let us denote by <*s*_1_,*s*_2_> the pair of semantic types of <*e*_1*i*_,*e*_2*j*_>. If and only if <*s*_1_,*s*_2_> exists in the Semantic Network, <*e*_1*i*_,*e*_2*j*_> is considered to constitute a relation.

SemRep also uses the Semantic Network in its extraction procedure. First, a predicate ontology was constructed by adding ‘indicator’ rules which map verbs and nominalizations to predicates in the Semantic Network; for example, ‘treat’ and ‘treatment’ are mapped to the predicate TREATS. Next, meta-rules that enforce the semantic types of the two arguments were also created on top of the indicator rules; an example of meta-rule is “Pharmacologic Substance TREATS Disease or Syndrome”. SemRep then matches predicates in text to these indicator rules and arguments’ semantic types to the meta-rules to identify relations. By using the ontology, SemRep can specify the name of the extracted relation, e.g., TREATS, AFFECTS, and LOCATION_OF, but limits itself in a fixed set of verbs. By contrast, PASMED is not restricted with a specific set of verbs, but it cannot assign a name to the extracted relation.

### Evaluating general relations

For the purpose of evaluation, we have created our original test set by randomly selecting 500 sentences from MEDLINE. Our system was given this set as input, and returned a set of binary relations as output. A binary relation in our setting is composed by two biomedical entities and it usually represents some association or effect between the entities. We call those binary relations *general* relations to distinguish them from those of specific types, e.g., PPI or DDI. To evaluate the general relations, we have defined evaluation criteria for entities and relations.

#### Evaluating entities:

**Figure 1:**
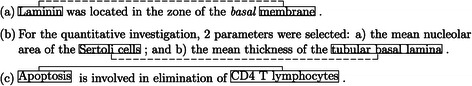
Examples of biomedical binary relations.**(a)** The relation is not correct because of one incorrect entity. **(b)** The relation is not correct because the relationship between the two entities is not represented explicitly by any semantic clue. **(c)** The relation is correct because it satisfies our two criteria of manually evaluation.

An entity is correct if and only if (1) it is a noun or a base noun phrase (a unit noun phrase that does not include other noun phrases), and (2) its content words represent the complete meaning within the sentence containing it. The first condition is set up in this criterion because MetaMap can only detect entities that are nouns or base noun phrases. The second one is to guarantee the meaning of the annotated entities. For example, Figure 1(a) shows a relation between two entities ‘Laminin’ and ‘membrane’. In this case, the entity ‘Laminin’ is correct, but the entity ‘membrane’ is not. The reason is that ‘membrane’ does not reflect the full meaning intended in this sentence; the right entity should be ‘basal membrane’.

#### Evaluating relations:

A correct relation must satisfy the following two conditions:

- The two entities composing the relation must be correct according to the above-mentioned criterion.
- The relationship between two entities in a correct relation must be described *explicitly* by some linguistic expression.

Any relations that break one of the above conditions are considered to be incorrect. For example, the extracted relation in Figure 1(c) is correct since it meets our criteria, while the extracted relations in (a) and (b) are not. The relation in (a) does not meet the first criterion since the entity ‘membrane’ is not correct. The relation in (b) does not meet the second criterion because this sentence only lists two selected parameters that are related to ‘Sertoli cells’ and ‘tubular basal lamina’, and no relationship between these two entities is mentioned. More details about our evaluation guideline can be seen in the Additional file 1.

## Results and discussion

In this work, we conducted evaluations in two scenarios: (1) extraction of all possible relations in sentences randomly sampled from MEDLINE, in which we attempt to estimate the performance of PASMED from a perspective of open-domain relation extraction from MEDLINE, and (2) extraction of relations predefined in PPI and DDI corpora.

### Evaluation results on general relations

For comparison, we conducted experiments using two state-of-the-art OIE systems for general domains, namely, ReVerb [11] and OLLIE [12]. We employed these two systems to extract relevant NP pairs in place of our PAS patterns. The other processes were applied in exactly the same way as our system. We also compared our system with the latest version of SemRep [40] on the test set.

Two annotators were involved in evaluating general relations. The two annotators, who are not co-authors of this article, have different backgrounds. Annotator A has a PhD in biology, majoring in genetics. Annotator B has a master degree of computer science, majoring in natural language processing; he is also a bachelor of medical biotechnology. The annotators were required to strictly follow our criteria when evaluating the outputs of the four systems: ReVerb, OLLIE, SemRep and PASMED. Both Annotator A and B were blind to the identity of the systems, i.e., they do not know which output was given by which system.

Both ReVerb and OLLIE assign a confidence value to each extracted triple instead of simply classifying them as true or false. In our experiments, this value was used as the threshold for extracting relations. We selected the values generating the best harmonic mean of precision and the number of true positives in our experiments, which turned out to be 0.7 for both systems. On our test set, ReVerb, OLLIE, SemRep and PASMED extracted 77, 164, 346, and 781 relations, respectively.

**Figure 2:**
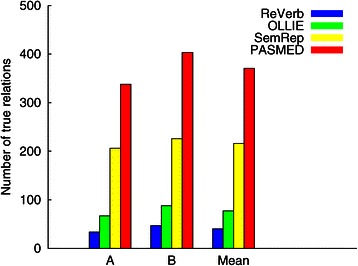
The number of true relations of the four systems on our test set according to the agreement of the two annotators. The mean numbers are 40.5, 77.5, 216, and 370.5, respectively. PASMED achieved the highest ones in all cases.

Figure 2 shows the numbers of true relations output by the four systems according to the two annotators. PASMED identified the highest number of true relations than the other systems. Specifically, the number of true relations extracted by PASMED was 71% higher than that of SemRep, which was the second best among the four systems. It should be noted that we can decrease the thresholds of ReVerb and OLLIE to increase their recalls. However, even when the thresholds were 0.3, their numbers of true positive relations were much lower than that of PASMED, which were about 52 and 103 on average, respectively.

In order to estimate the recall of these systems, we used *relative* recall defined by Clarke and Willett [41]. Let *a, b, c* and *d* denote the true relations of ReVerb, OLLIE, SemRep and PASMED respectively. We created a pool of gold-standard relations by merging *a, b, c, d* and removing duplicates. Let *r* denote the number of relations in the pool (*a*,*b*,*c*,*d*<*r*≤*a*+*b*+*c*+*d*), the recall of ReVerb is calculated as *a*/*r* and similarly for the other systems. We reported all scores of the four systems in Table 4. The higher recalls of PASMED in the table are in large part explained by the fact that the system has no restriction in predicate types, thereby accepting diverse biomedical relations. SemRep achieves a better precision score than PASMED by restricting the predicate types with its ontology but misses many relations due to the constraint. These results will be analyzed in more detail in the next section.

**Table 4:** **Evaluation results of the four systems according to the two annotators**

**System**	**Annotator A**			**Annotator B**			**Mean**		
	**Pre.**	**Re.**	**F.**	**Pre.**	**Re.**	**F.**	**Pre.**	**Re.**	**F.**
ReVerb	44.15	6.75	11.72	61.04	9.34	16.20	52.59	8.05	13.96
OLLIE	40.85	13.32	20.10	53.65	17.49	26.38	47.25	15.41	23.24
SemRep	59.37	40.95	48.47	65.13	38.83	48.65	62.25	39.89	48.56
PASMED	43.27	67.19	52.65	51.50	69.24	59.13	47.39	68.22	55.89

SemRep achieves the highest precision, PASMED achieves the highest relative recall.

A significance test on the F-scores of SemRep and PASMED was conducted by using approximate randomization [42]. We performed 1000 shuffles on the output of SemRep and PASMED and the approximate p-values according to the two annotators A and B are 0.35 and 0.02, respectively. These p-values indicate that with a rejection level of 0.05, there is a chance that the difference between SemRep and PASMED is statistically significant, which can be interpreted as the overall performance of PASMED is better than SemRep.

We have also calculated the Inter-Annotator Agreement (IAA) rates between the two annotators in each system by using *κ* statistics adapted to multiple coders [43]. We reports the values and their scales according to Green (1997) [44] in Table 5. The IAA scales indicate that the evaluation results are reliable enough.

**Table 5:** **The inter-annotator agreement rates between the two annotators in each system and their corresponding scale according to Green (1997) **[44]

**IAA**	**ReVerb**	**OLLIE**	**SemRep**	**PASMED**
	0.664	0.598	0.680	0.741
Scale	Good	Good	Good	Good

### Error analysis

We have listed the numbers of PASMED’s false positive relations caused by different types of errors in Table 6. On average, our system generated 410.5 false positive relations; among them (1) about 69.18% of them (284 false positive ones) are due to incorrect entitiy extraction (criterion 1), (2) 20.71% of false positive ones are not presented explicitly by linguistic expression (criterion 2) and (3) 10.11% break both criteria. The reason for the first case is that MetaMap occasionally fails to capture named entities with multiple tokens like the example in Figure 1(a). The second case is caused by parser errors and our greedy extraction. For instance, with this input “{[Laminin]}$\phantom {\dot {i}\!}_{{NP}_{1}}$ was *located in* {the zone of the basal [membrane], whereas [tenascin] was mainly found in the mucosal [vessels]}$\phantom {\dot {i}\!}_{{NP}_{2}}$”, based on the NP pair <*n*1,*N* *P*_2_> the system returned three relations: *r*_1_(Laminin, membrane), *r*_2_(Laminin, tenascin), and *r*_3_(Laminin, vessels). Among them, *r*_2_ and *r*_3_ break both evaluation conditions. In this example, the parser failed to detect the second NP of the pair; the correct one should be ‘the zone of the basal membrane’, not including ‘whereas’ clause. Then, from this incorrect pair, our greedy extraction generated *r*_2_ and *r*_3_ since we assume that every pair of entities in a NP pair constitutes a relation; even using the Semantic Network could not help in this case.

**Table 6:** **Numbers of false positive PASMED’s relations according to the two annotators**

	**C1**	**C2**	**Both**	**Total**
Annotator A	257	120	66	443
Annotator B	311	50	17	378
Mean	284	85	41.5	410.5
	69.18%	20.71%	10.11%	

We have classified them into three types of errors: C1–false positives caused by incorrect entity extraction (criterion 1), C2–false positives caused by not presented explicitly by linguistic expressions (criterion 2), and Both–false positives due to both C1 and C2.

As reported in the previous section, PASMED extracted much more relations than the other three systems. In the case of ReVerb and OLLIE, the main reason for their low performance is that these systems failed to capture NP pairs in many sentences. More specifically, ReVerb and OLLIE could not extract NP pairs from 150 sentences and 95 sentences respectively; our system could not extract pairs only from 14 sentences. Given the input sentence: “{[Total protein], [lactate dehydrogenase] (LDH), [xanthine oxidase] (XO), [tumor necrosis factor] (TNF), and [interleukin 1] (IL-1)}$\phantom {\dot {i}\!}_{{NP}_{1}}$ were *measured in* {[bronchoalveolar lavage fluid] (BALF)}$\phantom {\dot {i}\!}_{{NP}_{2}}$.”, ReVerb and OLLIE could not extract any triples, while our system generated a NP pair of <*N* *P*_1_,*N* *P*_2_> and returned five correct relations between ‘bronchoalveolar lavage fluid’ and five entities in *N* *P*_1_. This can be explained by the fact that these systems use general language parsers and those parsers do not perform well on biomedical texts, which contain more complex vocabularies and structures than the general one. In the case of SemRep, the main reason why it detected fewer relations than PASMED is that SemRep is restricted with a fixed set of verbs, which limits itself in a set of relations. For instance, SemRep also fails to extract relations in the above sentence because its ontology does not include the verb ‘measure’.

Since our PAS patterns focus on verbs and prepositions, there are relations that our system misses unlike SemRep, e.g., relations in the forms of modification/head of noun phrases. For example, SemRep identified a relation between ‘tumor’ and ‘malignancy’ in the sentence “Spontaneous [apoptosis] may play a role in evolution of [tumor] [malignancy]” while our system could not. It, instead, extracted the relation of (‘apoptosis’, ‘malignancy’) based on the phrase ‘play a role in’.

PASMED does not extract some relations that SemRep does since it filters MetaMap’s output. Given the sentence “We monitored a group of [*patients*] with [pollinosis] sensitive to Olea”. SemRep output a relation between ‘patients’ and ‘pollinosis’. PASMED ruled out ‘patients’ from MetaMap’s output at its filtering step because this entity is an overly frequent entity in MEDLINE.

Nevertheless, this filtering step helps our system to discard many spurious relations. For example, given the phrase “Morbidity risk for [alcoholism] and [drug abuse] in [*relatives*] of [cocaine addicts]”, two relations (‘relatives’, ‘alcoholism’) and (‘relatives’, ‘drug abuse’) were extracted by SemRep. The two annotators assessed these relations as incorrect on the ground that the word ‘relatives’ alone is not specific enough. By contrast, PASMED discarded ‘relatives’ because this entity is too frequent in MEDLINE. No relation composed by the entity was thus identified. Instead, PASMED detected two other relations, (‘alcoholism’, ‘cocaine addicts’) and (‘drug abuse’, ‘cocaine addicts’), which were assessed as correct by the annotators. We should note, however, that these relations are not strictly correct either, since the full description for the latter entity should be ‘relatives of cocaine addicts’.

As for the set of PAS patterns used in PASMED, it is not impossible to extend them to detect more relations. The maximal recall that could be reached is about 80% in the best case (the same recall of the GENIA corpus, see Table 2), but there is a higher risk that the precision will be decreased substantially due to three sources of errors, including MetaMap’s errors, parser’s errors and our greedy extraction. Currently, PASMED relatively covers 68.22% of general relations on average, which we deem to be high enough for the current trade-off.

Here we clarify the differences—besides the fact that PASMED uses deep syntax—between ReVerb, OLLIE, SemRep and PASMED, which are all based on a pattern-based approach. Regarding ReVerb and OLLIE, a major difference is that they employ a parser for the general domain while PASMED uses a parser specifically tuned for the biomedical domain. One of the biggest differences between SemRep and PASMED is the way the extracted relations are verified. SemRep restricts its relations using a predefined predicate ontology based on the Semantic Network. PASMED also depends on the Semantic Network but uses it in a less restrictive manner, which contributed to the system’s higher recall.

### Evaluation results on predefined relations

We also conducted experiments to see how well our PAS patterns cover predefined relations such as Protein-Protein Interaction (PPI) and Drug-Drug Interaction (DDI). Regarding PPI, we applied our patterns to AIMed, BioInfer and LLL–three popular corpora in this domain [3,33]. The gold-standard entities available in these corpora were used instead of MetaMap output. We conducted the same experiment for DDI on the SemEval-2013 task 9 corpus [7].

For comparison and reference, we show the precision and recall of some notable systems on PPI and DDI. It should be noted that since these systems used machine learning methods, they were evaluated by using 10-fold cross-validation or using the test set; while our method is pattern-based and thus we simply applied our patterns to the whole labeled corpora. The experimental results are shown in Tables 7 and 8. Quite expectedly, PASMED is outperformed by the supervised systems, although it shows comparable performance for the LLL corpus.

**Table 7:** **Performance of our system on AIMed, BioInfer and LLL corpora, compared with some state-of-the-art systems for PPI**

	**AIMed**		**BioInfer**		**LLL**	
	**Pre.**	**Re.**	**Pre.**	**Re.**	**Pre.**	**Re.**
Yakushiji et al. [2]	71.8	48.4	-	-	-	-
Airola et al. [3]	52.9	61.8	47.7	59.9	72.5	87.2
Miwa et al. [4]	55.0	68.8	65.7	71.1	77.6	86.0
PASMED	30.4	52.6	51.1	44.9	87.2	81.5

**Table 8:** **Performance of our system on MedLine and DrugBank corpora of SemEval-2013 Task 9 [** 7 **], compared with the highest and lowest-performing systems in that shared task**

	**MedLine**		**DrugBank**	
	**Pre.**	**Re.**	**Pre.**	**Re.**
Highest-performing system	55.8	50.5	81.6	83.8
Lowest-performing system	62.5	42.1	38.7	73.9
PASMED	27.0	62.5	41.0	61.6

**Figure 3:**
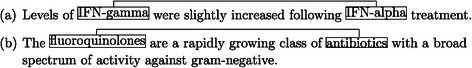
Examples of true extracted relations that are treated as false positive ones according to the annotated PPI and DDI corpora.**(a)** ‘associated_with’ relation. **(b)** ‘is_a’ relation.

Besides the parser’s errors and greedy extraction presented in the previous section, the seemingly low precision scores of PASMED are caused by the system’s generality. As stated before, our extraction schema covers any kinds of relations; it does not only focus on the interaction relationship. Therefore, even when the extracted relations are true, if they are not interaction relations, they are treated as false positives according to the gold-standard annotations. Figure 3 shows examples that PASMED extracted true relations between two proteins ‘IFN-gamma’ and ‘IFN-alpha’ in (a) and two drugs ‘fluoroquinolones’ and ‘antibiotics’ in (b), but their relationships are (a) ‘associated_with’ and (b) ‘is_a’, which are judged as false positives when compared with the annotated PPI and DDI corpora. We may improve the precision of our system by setting rules to filter out those kind of relations. For example, we can use a set of verbs that describe the relation of interaction, such as interact and activate, to validate the extracted relations.

**Figure 4:**
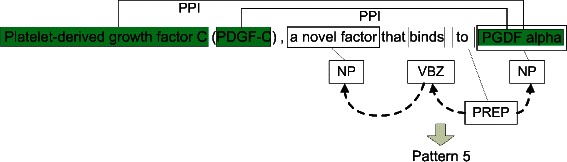
An example of two PPIs that need coreference information to be identified. Our system can detect a NP pair according to Pattern 5 but cannot extract any relations.

The low recall scores are due to the lack of patterns and coreference resolution. Figure 4 illustrates an example that our system missed two PPIs since it has no information about coreference that is essential to infer them. In this example, our system can detect a NP pair of (a novel factor, PGDF alpha) according to Pattern 5. The system, then, could not identify any relation since the first NP does not contain any entity. However, in fact, there are two PPIs between ‘PGDF alpha’ and the two coreferences of ‘a novel factor’, which are ‘Platelet-derived growth factor’ and ‘PDGF-C’. We have investigated 100 false negative PPIs on the AIMed corpus and found that there are 21 false negative ones (21%) caused by this error. It is clear that if PASMED could perform accurate coreference resolution, it would cover more interactions. Another solution would be to create more patterns to capture interaction expressions, such as ‘an interaction between A and B’, ‘a complex of/between A and B’, ‘A-B complex’, and ‘A-B binding’. There are 28 false negative interactions satisfying the expressions. However, these patterns are not general enough for all type relations; they are only specific for PPI and DDI.

### Semantic relations in MEDLINE

PASMED has been applied to the whole MEDLINE and extracted more than 137 millions of semantic relations in the format of (entity 1, relation phrase, entity 2). The ten most frequent types of relations are listed in Table 9. The most common semantic relation type is the relation between ‘Amino Acid, Peptide or Protein’ entities, which count up to 3.4 million. This explains partially why PPI has been attracting considerable attention in the BioNLP community. Many of the previous studies focus on improving PPI performance [3-5]. There are many large-scale databases constructed from MEDLINE focusing on PPI, e.g., MedScan [45], AliBaba [46], and Chowdhary et al. [47].

**Table 9:** **The ten most frequent types of semantic relations extracted from the whole MEDLINE**

**Rank**	**Semantic relation type**		**#Relation**	**#Unique**
	**Entity 1**	**Entity 2**		
1	Amino acid, Peptide or protein	Amino acid, Peptide or protein	**3,424,145**	1,057,771
2	Cell	Amino acid, Peptide or protein	3,140,492	711,603
3	Gene or genome	Amino acid, Peptide or protein	1,821,923	766,084
4	Disease or syndrome	Disease or syndrome	1,780,634	599,355
5	Body part, Organ, or Organ component	Amino acid, Peptide or protein	1,720,271	561,839
6	Amino acid, Peptide or protein	Disease or syndrome	1,621,104	631,343
7	Gene or genome	Cell	1,142,425	315,794
8	Organic chemical	Organic chemical	1,122,133	365,631
9	Body part, Organ, or Organ component	Body part, Organ, or Organ component	1,119,095	270,886
10	Laboratory procedure	Amino acid, Peptide or protein	1,109,260	453,359

Another type of relation that is also extensively studied in the community is the relation between genes and proteins, which is ranked third in Table 9. As with PPI, there are many studies and databases related to this type of relations, such as Chilibot [48], MEDIE [49], EVEX [50] and the BioNLP Shared Task [9].

The second most common type of relations in our extraction result is the ones between cell and protein entities, which appeared more than 3.1 million times in MEDLINE. This type of relations contain many localization and whole-part relations, the information of which is potentially very useful in biology. These relations are covered partially by *localization events* in the GENIA corpus. The events are represented as ‘Localization of Protein to Location’ where Location can be cells. Recently, the CG task [51] has also targeted events on ‘Localization of Proteins at/from/to Cells’.

Somewhat unexpectedly, the relations between genes and diseases, which are another important type of biomedical relations [52], turned out to be much less common than PPIs. More specifically, its rank was the 41 ^*th*^ and the number of relations extracted from MEDLINE was about 583,000.

The last column in Table 9 shows that the diversity of the semantic relations is slightly different from their occurrences. For instance, the cell-protein relations are more frequent but less diverse than the gene-protein ones.

## Conclusion

In this work, we have developed PASMED to extract diverse types of relations between biomedical entities from the literature. Six simple but effective PAS patterns have been proposed to detect relevant NP pairs. Our evaluation results have confirmed that our pattern-based system covers a wide range of relations. Although the precision scores of PASMED fell short of those of SemRep, the overall results suggest that PASMED compares favorably with SemRep, extracting a significantly higher number of relations.

We have applied PASMED to the entire MEDLINE corpus and extracted 137 million semantic relations. This large-scale and machine-readable output can be used to scale-up high-quality manual curation of a relation ontology or served as a knowledge base for semantic search.

For future work, we plan to extend our system to address *n*-ary relations [53,54]. Relations of this type are more informative than binary ones since they can include details about the site, context or conditions under which biomedical relations occur.

## Availability of supporting data

The data sets supporting the results of this article are available in the PASMED repository: http://www.logos.t.u-tokyo.ac.jp/\%7Enhung/PASMED/.

## Additional file

Additional file 1
**Evaluation Guideline.** A.pdf file that presents details about our guideline to evaluate general relations in our setting. The guideline gives more examples to demonstrate the criteria of evaluating relations. It explicitly describes some exceptions that the annotators must follow during their evaluation process.
