# Supplementary material for: Wide-coverage relation extraction from MEDLINE using deep syntax
Source: BMC Bioinformatics. 2015 Apr 1;16:107. doi: 10.1186/s12859-015-0538-8 (PMC4396593; doi:10.1186/s12859-015-0538-8)
Supplement: Additional file 1 — Evaluation Guideline. A.pdf file that presents details about our guideline to evaluate general relations in our setting. The guideline gives more examples to demonstrate the criteria of evaluating relations. It explicitly describes some exceptions that the annotators must follow during their evaluation process. [file 12859_2015_538_MOESM1_ESM.pdf]

Guideline for Evaluating Biomedical Binary Relations

Nhung T. H. Nguyen[1] and Yoshimasa Tsuruoka[2]

[1] School of Information Science
Japan Advanced Institute of Science and Technology, Japan
nthnhung@jaist.ac.jp
[2] Graduate School of Engineering
The University of Tokyo, Japan
tsuruoka@logos.t.u-tokyo.ac.jp

1. Biomedical binary relations

In our scenario, biomedical binary relations, composed by two biomedical entities, show associations or effects between the entities. For instance, in Figure 1-(a), there is one binary relation between 'Apoptosis' and 'CD4 T lymphocytes', which indicates that 'Apoptosis' somehow affects 'CD4 T lymphocytes'. Figure 1-(b) presents three relations r_1(heart, camels), r_2(Purkinje cells, collagen fibres) and r_3(Purkinje cells, connective tissue), these relations tell us that there are associations between these entities. The associations can be of any type, such as 'part-of', 'separated by', and 'surrounded by' relations.

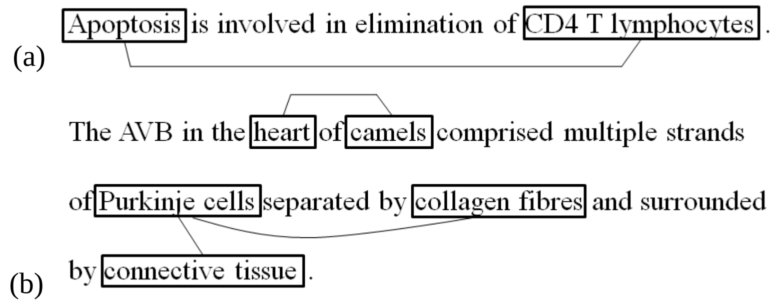

Figure 1. Examples of biomedical relations.

2. Evaluation principles

We define two principles for evaluating binary relations in our scenario. The first one is related to the entities' boundaries, and the second one is about the syntactic structure that represents the semantic relation in a sentence.

2.1 Evaluating entities

Entities in our setting are nouns or base noun phrases in sentences. An entity is correct if and only if its content words represent the complete meaning within the sentence containing it.

Example 1: Alterations in the microcirculatory bed of the thalamus resulting from thermal trauma ...

Entity	Correct?	Comments
microcirculatory bed	Yes	
thalamus	Yes	
trauma	No	It should be 'thermal trauma'

In example 1, the first two entities 'microcirculatory bed' and 'thalamus' are correct, but the entity ‘trauma’ is NOT. The reason is that ‘trauma’ does not reflect the complete meaning intended in this sentence; the right one should be ‘thermal trauma’.

Follows are some rules applied to some specific cases.

2.1.1 Rule 1 for discontinuous entities

- It should be noted that in biomedical text, sometimes, entities appear in *discontinuous text regions*. For instance, given the following sentence:

  Example 1a: We investigated spontaneous and lipopolysaccharide (**LPS**) stimulated production of **tumor necrosis factor alpha** (TNF alpha), **interleukin** (IL) **1**, **IL-6**, and **IL-8** .

Entity	Correct?	Comments
LPS	Yes	
tumor necrosis factor alpha	Yes	
interleukin 1	Yes	Correct even though it's discontinuous
IL-6	Yes	
IL-8	Yes	

All extracted entities are correct despite the fact that 'interleukin 1' is discontinuous.

- There are cases in which discontinuous entities are not correct, such as entity 'interventricular septum' in example 1b. This entity is not correct, it should be 'interventricular membranous septum'.

  Example 1b: The atrioventricular bundle entered the lower part of the interventricular membranous septum ...

Entity	Correct?	Comments
atrioventricular bundle	Yes	
interventricular septum	No	It should be 'interventricular membranous septum'

2.1.2 Rule 2 for noun modifiers

- An entity is "correct" even if it fails to include the common nouns or head nouns located at the end of the phrase, as long as the entity conveys the main meaning of the phrase. For instance, entities 'probiotic' in example 2a is correct since its meaning is sufficient without including the word 'effects'. More specifically, in the noun phrase 'probiotic effects', 'effects' is the head noun and modified by 'probiotic', which is a bacteria name that expresses the main meaning of this phrase. Therefore, 'probiotic' can be correct in our setting. The same explanation applies to 'ciguatera' in example 2b.

  Example 2a: Saccharomyces boulardii is a strain of yeast which has been extensively studied for its probiotic effects.

Entity	Correct?	Comments
Saccharomyces boulardii	Yes	
probiotic	Yes	'probiotic' is equal to 'probiotic effects'
yeast	Yes	

Example 2b: Ciguatoxins, the principal causative toxins of ciguatera seafood poisoning, are large ladder-like polycyclic ethers.

Entity	Correct?	Comments
Ciguatoxins	Yes	
toxins	Yes**	According to rule 2b
ciguatera	Yes*	since 'ciguatera' itself also includes the meaning of 'seafood poisoning'
poisoning	No	'ciguatera seafood poisoning'
ethers	No	It should be 'large ladder-like polycyclic ethers' or 'ladder-like polycyclic ethers'

- If the extracted entities are common nouns or head nouns, they are not correct. For example, 'progastrin' in the following sentence is the head of noun phrase 'tissue progastrin' and modified by 'tissue'. It is not correct since in this context it should include the modifier 'tissue' to be specific enough.

  Example 2c: ... tissue progastrin was elevated by only about 50%.

Entity	Correct?	Comments
progastrin	No	It should be 'tissue progastrin'

2.1.3 Rule 3 for adjective modifiers

- Ideally, adjectives/adjective phrases that modify nouns/noun phrases should be included in the extracted entities. However, if the adjectives/adjective phrases are *general* ones, such as 'large', 'excessive', 'principal', and 'causative', they can be excluded from the entities. For instance, entity 'toxins' in example 2b is correct even though it does not include the adjective phrase 'principal causative'. Entity 'selenium' in example 3c is also correct without the adjective 'excessive'.
- In contrast, if that adjective or adjective phrase presents a *biological meaning*, it must be included in the entity, such as in example 1, the adjective 'thermal' must be included in 'thermal trauma' to make its meaning complete. This rule is demonstrated in the following examples.

  Example 3a: The atrioventricular bundle ran through the *fibrous* trigone ...

Entity	Correct?	Comments
atrioventricular bundle	Yes	
Trigone	No	It should be 'fibrous trigone'

  Example 3b: ... was investigated in thirty *prepubertal* children.

Entity	Correct?	Comments
Children	No	It should be 'prepubertal children'

  Example 3c: Laminin is located in the zone of the *basal* membrane.

Entity	Correct?	Comments
Laminin	Yes	
Membrane	No	It should be 'basal membrane'

2.1.4 Rule 4 for possessive forms

- If there is a preposition 'of' between two entities to show their part-whole relation, and these two entities have sufficient meaning, they are correct. For example, entities 'heart' and 'camels' in the following sentence are correct, even though the proper entity should be 'heart of camels'. The same explanation applies to 'strands' and 'Purkinje cells'.

  Example 4a: the AVB in the heart of camels comprised multiple strands of Purkinje cells ...

Entity	Correct?	Comments
Heart	Yes	Even though the proper one is 'heart of camels'
Camels	Yes	Even though the proper one is 'heart of camels'
Strands	Yes	Even though the proper one is 'strands of Purkinje cells'
Purkinje cells	Yes	Even though the proper one is 'strands of Purkinje cells'

- Strictly speaking, in example 4b, the entity 'rhesus monkeys' should be 'responses of rhesus monkeys', which is more specific and accurate in this context. However, in our

setting, 'rhesus monkeys' is acceptable because we can infer from the sentence that somehow there is a vague relation between 'rhesus monkeys' and 'pentobarbital', and we would like to extract such vague relations also. Entity 'vitamin E deficiency' in example 4c also demonstrates this exception.

Example 4b: Responses of **rhesus monkeys** were reinforced by delivery of either a **pentobarbital** (4.0 mg/ml) *solution* or a vehicle (water) or **saccharin** *solution* under a concurrent signaled differential reinforcement of low rates 30-s schedule.

Entity	Correct?	Comments
rhesus monkeys	Yes*	'responses of rhesus monkeys'
pentobarbital	Yes	According to rule 2a
saccharin	Yes	According to rule 2a

Example 4c: An excessive selenium supply compensated to a great extent for the effects of vitamin E deficiency on IgG and IgA.

Entity	Correct?	Comments
selenium	Yes	According to rule 2b
vitamin E deficiency	Yes*	'effects of vitamin E deficiency'
IgA	Yes	

2.2 Evaluating extracted relations

A correct relation must satisfy the following two conditions:

- P1: The two entities composing the relation must be correct according to the above-mentioned criteria.
- P2: The semantic relationship between two entities in the relation must be represented explicitly by some *syntactic structures* of the sentence.

Any relations that break one of the above two conditions are incorrect.

For example, all extracted relations in Figure 1 are correct since they satisfy our criteria. All extracted entities are correct; their semantic relationship are presented explicitly such as "[Apoptosis] ... involved in ... [CD4 T lymphocytes]"; "... [Purkinje cells] separated by [collagen fibres] ..."

In contrast, all extracted relations in Figure 2 are not correct. The two relations r1 and r4 break condition 1 since the entities 'membrane' and 'vessels' are not correct. Relation r2 breaks condition 2 because this sentence has two clauses: one is about 'Laminin' and the other is about 'tenascin', and there is no information to show their semantic relationship. Relation r3 breaks both conditions because entity 'vessels' is not correct and the relationship between 'Laminin' and 'vessels' is not presented in this sentence.

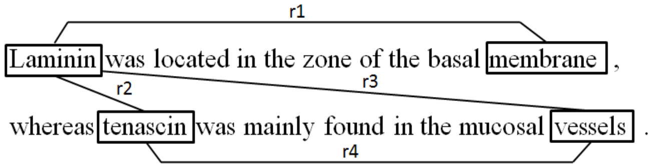

Figure 2. Examples of extracted relations that do not satisfy the two evaluation principles. r1 and r4 break principle 1; r2 breaks principle 2; r3 breaks both principle.

For illustrating condition 2 clearly, we have listed more examples of extracted relations that break this condition in Table 1.

No.	Extracted relations	Evaluation
1	Age-standardised adult diabetes prevalence was 9.8% (8.6-11.2) in men and 9.2% (8.0-10.5) in women in 2008, up from 8.3% (6.5-10.4) and 7.5% (5.8-9.6) in 1980.	The relation between 'men' and 'women' is incorrect since their semantic relationship is not mentioned in this sentence.
2	The PBR bind with high affinity the ligands Ro 5-4864 and PK 11195, but not clonazepam, which binds with high affinity to central-type benzodiazepine receptors (CBR).	This extracted relation is incorrect because of two reasons. Firstly, it breaks condition 1 since entity 'benzodiazepine receptors' is incorrect. Secondly, this sentence discusses two independent topics, one is 'PBR' and the other is 'clonazepam'.
3	The immunoglobulins of type IgA, IgM and IgG with the subtypes IgG1 , IgG2a, IgG2b and IgG2c were measured by immunoelectrophoresis.	All four extracted relation are incorrect because they breaks condition 2. We can see that 'IgG1', 'IgG2a', 'IgG2b' and 'IgG2c' are subtypes of 'IgG', and this sentence lists 'IgA' and 'IgG' but says nothing about their relation. Therefore, there is no relationship between 'IgA' and 'IgG' subtypes.
4	"Ferrum", a ferric hydroxide sucrose complex used clinically for iron deficiency anemia for more than 40 years, was investigated as a negative MRI contrast agent in five rabbits bearing experimental PE as well as in five normal volunteers.	The relation between 'iron deficiency anemia' and 'rabbits' is incorrect. We can infer from this sentence that 'Ferrum' was used for two independent purposes. One is related to 'iron deficiency anemia', and the other is related to 'negative MRI contrast agent in rabbits'. However, this sentence does not mention the relationship between these purposes.
5	For the quantitative investigation 2 parameters were selected: a) the mean nucleolar area of the Sertoli cells ; and b) the mean thickness of the tubular basal lamina .	This relation is incorrect since it breaks both conditions. Firstly, entity 'basal lamina' is not correct; it should be 'tubular basal lamina'. Secondly, this sentence lists two selected parameters that are related to 'Sertoli cells' and 'tubular basal lamina', but no relationship between them is mentioned.

Table 1. Examples of extracted relations that are incorrect because their semantic relationships are not shown in the sentence.

❖ <u>Exception 1</u>: there are some cases where the relation between two entities is not directly shown by the syntactic structure, but if that relation can be inferred through the sentence, *it can be assessed as a TRUE relation*. The example in figure 3 illustrates this case.

The system extracts nine relations; three of them, represented by solid lines, are correct since we can see the syntactic clues very clearly. The relation between 'heart' and 'Purkinje cells', represented by a dash line, is inferred based on the following reasoning: 'the AVB' is a part of the 'heart', 'the AVB' comprises 'strands of Purkinje cells', therefore 'heart' and 'Purkinje cells' most likely have some relations. The other five indirectly relations can be inferred in the same way.

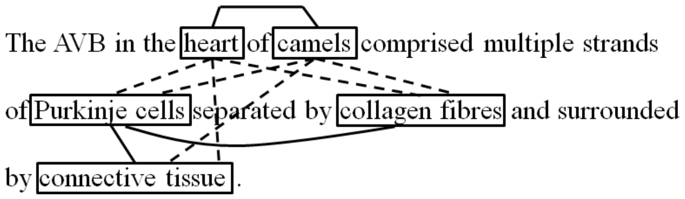

Figure 3. An example of indirect relations (dot lines). These relations are not directly represented through the syntactic structure but can be inferred based on syntactic clues.

3. The output's format

A test set including 500 sentences randomly selected from MEDLINE[1] was given to four different systems. These systems returned a set of binary relations as output. Each binary relation is presented in four fields consisting of (1) the start position of the first entity, (2) the first entity, (3) the start position of the second entity and (4) the second entity in a sentence, as shown in Table 2.

Start1	Entity 1	Start2	Entity 2	TRUE/FALSE	Comments
**Sentence 1**: The atrioventricular bundle ran through the fibrous trigone and entered the lower part of the interventricular membranous septum, beneath the right endocardium, then lay over or slightly to the side of the centre of the muscular interventricular crest.					
4	atrioventricular bundle	52	trigone	FALSE	P1
4	atrioventricular bundle	148	endocardium	FALSE	P1
4	atrioventricular bundle	94	interventricular septum	FALSE	P1
4	atrioventricular bundle	246	crest	FALSE	P1
**Sentence 2** :The detection of the illegal use of clenbuterol (CBL) as a growth promoter has relied on detecting residual concentrations of the drug in body fluids or tissues.					
36	clenbuterol	138	body fluids	TRUE	
36	clenbuterol	153	tissues	TRUE	

Table 1. Samples of the output and the evaluation of binary relations, the two final columns are filled by annotators.

4. Tasks for annotators

The annotators are required to:
- Evaluate all binary relations extracted from the 500 sentences by the four systems.
- Strictly follow our guideline to assess the extracted relations:
    - Extracted relations that satisfy the two conditions are TRUE,
    - otherwise they are FALSE.

When the evaluators assign FALSE to a relation, please specify which condition is not satisfied. If it breaks the first condition, please write 'P1' in the column 'Comments'. If it breaks the second one, please write 'P2'. If it breaks both, please write 'both'. Otherwise, please tell us your opinion.

In case of exception 1 in section 2.2, if the annotators assign FALSE to indirect relations and the reason is not P1, they have to explain their reasoning clearly.

In case the annotators do not follow any rules or principles, please clarify the reason.

[1] The version used in our system is the 2012 MEDLINE/PubMed baseline database http://www.nlm.nih.gov/bsd/licensee/2012_stats/baseline_med_filecount.html
